# Trastuzumab use in older patients with HER2-positive metastatic breast cancer: outcomes and treatment patterns in a whole-of-population Australian cohort (2003–2015)

**DOI:** 10.1186/s12885-019-6126-y

**Published:** 2019-09-11

**Authors:** Benjamin Daniels, Belinda E. Kiely, Monica Tang, Hanna Tervonen, Sallie-Anne Pearson

**Affiliations:** 1 Medicines Policy Research Unit, Centre for Big Data Research in Health, University of New South Wales (UNSW), Lowy Cancer Research Centre, Kensington, NSW 2052 Australia; 2 NHMRC Clinical Trials Centre, University of Sydney, Sydney, Australia

**Keywords:** HER2-positive, Metastatic breast cancer, Trastuzumab, HER2-targeted therapy, Older, Elderly, Population-based

## Abstract

**Background:**

Older patients with HER2-positive metastatic breast (HER2 + MBC) cancer are underrepresented in clinical trials. We aim to describe the treatment patterns and overall survival (OS) for older women receiving trastuzumab for HER2 + MBC.

**Methods:**

Retrospective, whole-of-population cohort study using demographic, dispensing, and medical services data for Australian women ≥ 65 years initiating trastuzumab for HER2 + MBC between 2003 and 2015. We describe time-on-trastuzumab; type and timing of other cancer treatments; rates of cardiac monitoring; and OS from trastuzumab initiation for HER2 + MBC.

**Results:**

Of 5404 women initiating trastuzumab for HER2 + MBC, 1583 (29%) were ≥ 65 years old, and the proportion of older patients increased from 20% in 2003 to 38% in 2015. The median age for older women was 73 years and 516 (33%) were ≥ 75 years. Most older patients (92%) received ≥3medicines for comorbidities other than cancer. Median (IQR) time on trastuzumab was 14.1 months (5.9–32.1) and on all chemotherapy was 5.6 months (3.3–10.8). 74% received ≥1 chemotherapy agent and 56% received endocrine therapy. Half (49%) of patients had a cardiac assessment prior to initiating trastuzumab and overall 1228 (76%) had ≥1 cardiac assessment during the study period. At a median follow-up of 6 years, 73% of patients had died and the median OS was 25.6 months (IQR 10.7–58.7).

**Conclusions:**

Older patients comprise a growing proportion of patients treated with HER2-targeted therapies in the real-world but they remain underrepresented in trials of these agents. Few trials report duration or OS estimates for older patients but our estimates are similar to those from trials that have. Although cardiac monitoring was a requirement of accessing trastuzumab during our study period, many patients did not undergo a cardiac assessment.

## Background

Evidence generated in randomized controlled trials (RCT) about the benefits and risks of new breast cancer treatments forms the basis of treatment guidelines, regulatory decisions, and communications between clinicians and patients about their likely prognosis and experience while undergoing treatment. Because RCTs are conducted in selected patient populations under standardized conditions, trial efficacy and safety outcomes may not directly apply to the more heterogeneous populations treated in routine clinical practice [1, 2]. Notably, trials seldom include older participants in proportions representative of the real-world clinical population. For example, 43% of newly diagnosed breast cancers occur in people over 65 years of age [3–5], but the proportion of participants ≥ 65 in breast cancer clinical trials has been estimated at just 9% [6]. Evidence around the efficacy and safety of new treatments is, therefore, largely absent for a substantial proportion of patients undergoing breast cancer treatment.

In the case of HER2-positive metastatic breast cancer (HER2 + MBC), treatment patterns and survival outcomes are not well understood in older patients. Pivotal trials of HER2-targeted agents indicate that survival estimates have been increasing over time [7–9], however, the trend in survival over time for older patients is unknown [9, 10], with most RCTs including only a small number of participants ≥65. For example, in recent HER2 + MBC trials, approximately 15% of participants were ≥ 65 and only 2–3% were ≥ 75 [11–13]. In the original pivotal trial, which evaluated trastuzumab (Herceptin, Genentech, South San Francisco, CA; Hoffmann-La Roche Ltd., Basel, Switzerland) plus chemotherapy against chemotherapy alone, 23% of patients were > 60 and the oldest participant in the trial was 77 [7, 10]. Sub-group analysis of these patients found that they received benefit in terms of overall survival (OS) from the addition of trastuzumab to chemotherapy, but rates of cardiotoxicity were double those reported in patients ≤ 60 [10].

More recently, the CLEOPATRA trial, evaluating first-line pertuzumab (Perjeta, Genentech, South San Francisco, Ca; Hoffmann-La Roche Ltd., Basel, Switzerland), trastuzumab, and chemotherapy against trastuzumab plus chemotherapy, found the addition of pertuzumab was associated with increased progression-free survival (PFS) and OS in patients ≥65 (16% trial participants). However, there were no differences between treatment arms for patients ≥75 (2% of trial participants) [8, 9]. The incidence of cardiotoxic events was low and similar between older and younger patients in the CLEOPATRA trial [9].

The majority of observational research in older patients with HER2+ breast cancer has focused on early-stage disease [14]. There are few data from observational studies on the treatment and outcomes for older patients with HER2 + MBC and in the present study we sought to describe the real-world treatment patterns and survival outcomes for patients ≥ 65 starting trastuzumab for HER2 + MBC.. Our specific objectives were to determine the: time-on-trastuzumab and other HER2-targeted therapies; time-on-other cancer therapies; patterns of cardiac monitoring; and OS in this older population.

## Methods

### Setting and data

The Australian healthcare system and the datasets we used in our study have been described previously in our research protocol [15]. Briefly, Australia maintains a publicly funded, universal healthcare system entitling all citizens and permanent residents to subsidized medicines through the Pharmaceutical Benefits Scheme (PBS) and subsidized medical services through the Medicare Benefits Schedule (MBS). The *Herceptin Program*, separate to the PBS, provided fully subsidized access to trastuzumab for HER2 + MBC from December 2001 until July 2015, when the program was closed and trastuzumab for HER2 + MBC was listed for subsidy on the PBS [15]. Lapatinib was subsidized by the PBS from May 2008 while pertuzumab and trastuzumab emtansine (T-DM1; Kadcyla, Genentech, South San Francisco, Ca; Hoffmann-La Roche Ltd., Basel, Switzerland) were subsidised from July 2015 [15].

The Australian Department of Human Services (DHS) – administering body for the *Herceptin Program* and PBS– supplied de-identified, patient-level data including: patient demographic information, HER2 testing results (immunohistochemistry (IHC) or in-situ hybridization (ISH)), records of trastuzumab dispensed to *Herceptin Program* enrollees, and all PBS dispensing records for *Herceptin Program* enrollees [15]. The DHS also provided the dispensing records for all patients in Australia accessing publicly-subsidized trastuzumab for early breast cancer (EBC) from 1 October 2006 to 30 June 2016. We determined previous treatment with trastuzumab for EBC through data linkage of *Herceptin Program* records with the dispensing records of patients who received trastuzumab for EBC.

The period of time observed across the datasets is 1 January 2001 to 30 June 2016.

### Study design and participants

Our population-based, retrospective cohort study includes every Australian woman initiating trastuzumab for MBC subsidized through the *Herceptin Program* between 1 January 2003 and 30 June 2015. We considered older patients to be those initiating trastuzumab at age 65 or older. Our data collection is limited by a lack of clinico-pathological information and thus we have chosen not to make direct comparisons between older and younger populations in our study as we could not adjust for important confounders. However, we provided outcome estimates for patients < 65 for descriptive context. All patients were observed from initiation of trastuzumab for MBC until death or 30 June 2016.

### Outcomes and statistical analysis

We used descriptive statistics to summarize age, weight, the number and proportion of patients who died, and determination of HER2+ status (IHC or ISH). We used a validated algorithm, applied to the dispensing records from up to 1 year prior to initiation of trastuzumab for MBC for older patients, to identify treatment for comorbid disease [16]. PBS dispensing records contain all dispensed medicines for patients ≥65 years and these records were used to estimate patient comorbidities. Patients < 65 are required to pay a co-payment towards prescribed medications which ranged from $23.00 AUD in 2003 to $37.70 AUD in 2015. As PBS data are collected for the primary purpose of providing financial reimbursement from the federal government, medicines costing below this co-payment amount do not attract government reimbursement and, therefore, do not appear in PBS dispensing records because the patient bears the full cost of the medicine [17]. For this reason, we cannot be sure we observe all of the medicines dispensed to patients < 65 and we restricted our estimates of comorbid treatments to patients ≥ 65.

We estimated: OS from the time of first trastuzumab dispensing for HER2 + MBC until month of death (set at the last day of the month) or censor and; time on treatment as the period of time from first observed dispensing date of each therapy until the sooner of either the last observed dispensing date plus 30 days or the number of days to death [18, 19]. We considered a period of > 90 days between dispensings as a break in a course of treatment and a subsequent dispensing following a break of > 90 days as beginning a new course of therapy [20].

We used PBS dispensing data to summarize the type, number, and timing of other cancer treatments dispensed following trastuzumab initiation for HER2 + MBC. We determined first-line partner therapy based on treatments dispensed during the period from 30 days prior to 90 days following trastuzumab initiation. We calculated the time on other cancer therapies in the same manner as for HER2-targeted therapies, and summarized the number of unique chemotherapies dispensed following trastuzumab initiation.

For each patient we used MBS claims to identify the number of cardiac assessments (echocardiography and gated cardiac blood pool scans) and when they were performed. We defined baseline cardiac assessments as those assessments with a claim date from 60 days prior, to 30 days following the date of first dispensing of trastuzumab. We defined on-treatment cardiac assessments as those claimed from 30 days after the first dispensing until the end of the trastuzumab course [18].

All analyses were performed in SAS version 9.4 (SAS Institute, Cary, NC) with figures generated in R version 3.3.5.

## Results

### Patient characteristics

**Fig. 1:**
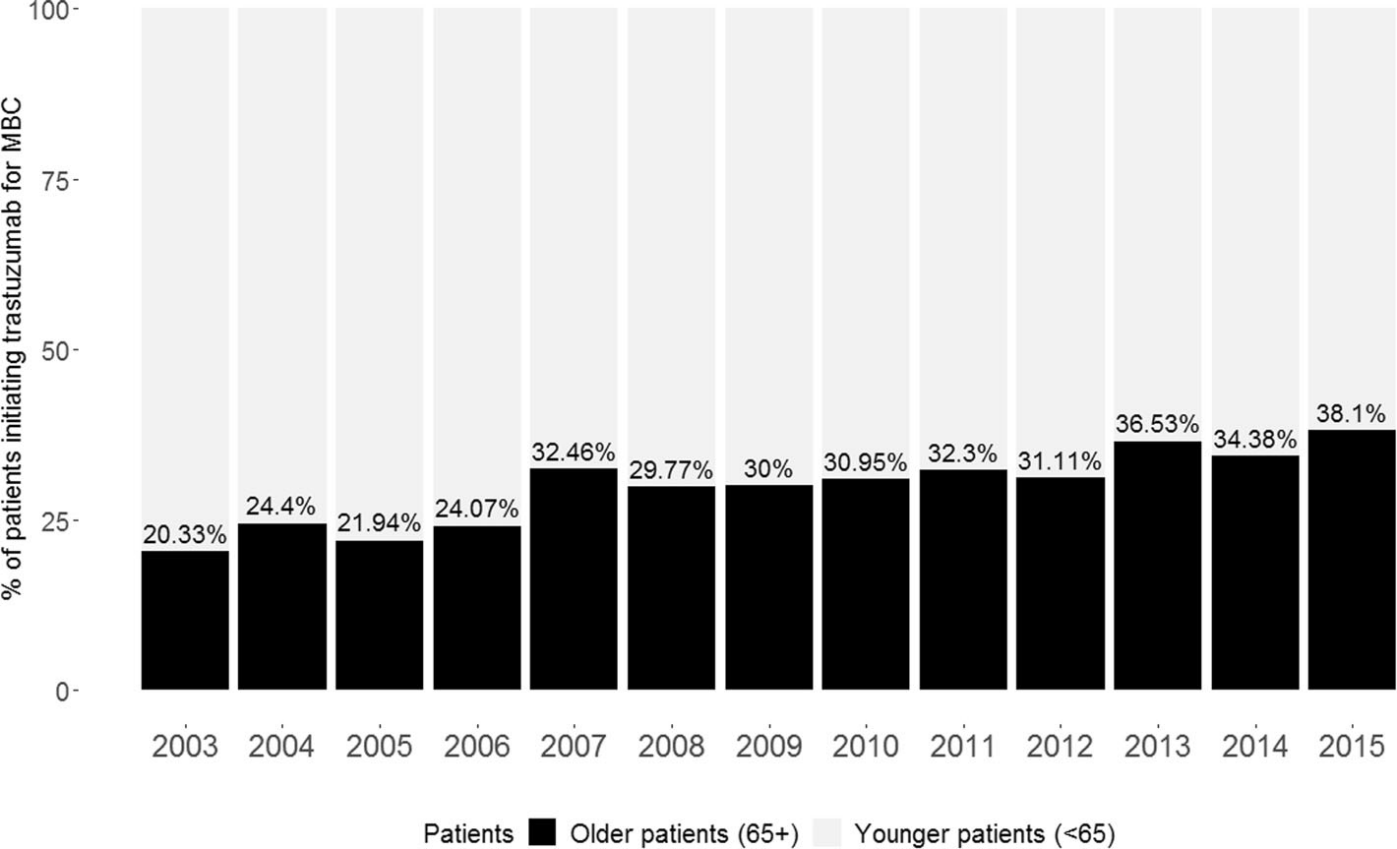
Proportion of older patients initiating trastuzumab for MBC in each year

Of the 5404 women initiating trastuzumab for HER2 + MBC between 3 December 2001 and 30 June 2015, 1583 (29%) were ≥ 65. The median age for this older cohort was 73 years (IQR: 68–78), with 516 (10%) ≥ 75 and 130 (2%) ≥85. A minority of older women (189; 12%) had received adjuvant trastuzumab. Most (1458; 92%) had received three or more medicines to treat comorbid conditions prior to initiating trastuzumab (Table 1). The proportion of older patients initiating trastuzumab for MBC increased steadily over the study period, from 20% of all patients initiating trastuzumab for MBC in 2003 to 38% in 2015 (Fig. 1, Additional file 1: Figure S1B). The median follow-up time for older patients was 5.7 years (IQR: 3.3–8.8).

**Table 1:** Characteristics of patients 65 years and older, those younger than 65 years, and the entire cohort

	Older patients (≥ 65)	Younger patients (< 65)	All patients
Patients (n)	1583	3821	5404
Baseline measures			
Age in years at first metastatic trastuzumab dispensing, median (IQR)	73 (68–78)	52 (45–58)	57 (48–66)
< 35	–	138 (4%)	138 (3%)
35–44	–	760 (20%)	760 (14%)
45–54	–	1372 (36%)	1372 (25%)
55–64	–	1551 (40%)	1551 (29%)
65–74	937 (59%)		937 (17%)
75–84	516 (33%)	–	516 (10%)
85+	130 (8%)	–	130 (2%)
Weight (kgs) at *Herceptin Program* enrolment, median (IQR)	68 (59–77)	70 (60–82)	70 (60–80)
Treatment qualification:			
HER2-positive by IHC^a,c^ 3+, n	871 (55%)	2285 (60%)	3156 (58%)
HER2-positive by ISH^b,c^, n	734 (46%)	1615 (42%)	2349 (43%)
Previously treated with trastuzumab for EBC, n	189 (12%)	612 (16%)	801 (15%)
Number of medicines dispensed to treat comorbidity, n patients:^d^			
No medicines	52 (3%)	–	–
1–2 medicines	73 (5%)	–	–
3 or more medicines	1458 (92%)	–	–
Treatment with at least one cardiovascular medicine in the year prior to trastuzumab initiation^e^, n	1047 (66%)	–	–
Post-trastuzumab initiation measures			
Deaths, n	1153 (73%)	2622 (69%)	3775 (70%)
Median follow-up time^f^, years (IQR)	5.7 (3.3–8.8)	6.7 (3.9–10.3)	6.4 (3.8–9.9)

^a^Immunohistochemistry

^b^In-situ hybridisation

^c^Patients may have multiple tests

^d^Number of comorbidities assessed using the *RxRisk* algorithm. For patients younger than 65 years of age, medicines costing below the PBS co-payment threshold are not captured in the data and a comorbidity estimate cannot be determined for these patients

^e^Cardiovascular medicines are those whose ATC code begins with “C”

^f^Median follow-up time calculated according to the reverse Kaplan-Meier method

### HER2-targeted therapy

Median time on trastuzumab for HER+MBC, excluding breaks, was 14.1 months (IQR: 5.9–31.1; Table 2) and the median duration of the first course of trastuzumab was 12.1 months (IQR: 4.6–24.9; Table 2). More than half of older patients (838; 53%) continued trastuzumab beyond 1 year and 82 patients (5%) continuously received the medicine for more than 5 years. Trastuzumab was started as monotherapy for 23% of older patients (Table 3), and trastuzumab was the only cancer therapy dispensed to 10% of older patients (Table 2).

**Table 2:** Trastuzumab, HER2-targeted therapy, chemotherapy, and endocrine therapy treatments and durations

	Older patients (> 65)	Younger patients (< 65)	All Patients
Patients, n	1583	3821	5404
Trastuzumab			
Number of trastuzumab courses,^a^ n patients			
One course	1583 (100%)	3821 (100%)	5404 (100%)
Two courses	319 (20%)	1079 (28%)	1398 (26%)
Three or more courses	61 (4%)	341 (9%)	402 (7%)
Median time on all trastuzumab treatment,^b^ months (IQR)	14.1 (5.9–32.1)	18.2 (8.7–40.9)	16.8 (7.8–38.2)
Median duration of the first uninterrupted course of trastuzumab, months (IQR)	12.1 (4.6–24.9)	14.0 (6.5–28.9)	13.5 (5.9–27.6)
Trastuzumab monotherapy only, n	154 (10%)	163 (4%)	317 (6%)
HER2-targeted therapies			
Treated with other HER2-targeted therapies for MBC, n:	340 (21%)	1404 (37%)	1744 (32%)
Lapatinib	182 (11%)	950 (25%)	1132 (21%)
Pertuzumab	91 (6%)	284 (7%)	375 (7%)
T-DM1	101 (6%)	300 (8%)	401 (7%)
Median time on all HER2-targeted therapies,^b^ months (IQR)	15.4 (6.3–37.0)	21.5 (10.1–48.8)	19.4 (8.8–45.0)
Median proportions of observed survival time^c^ on all HER2-targeted therapies, including trastuzumab, % (IQR)	85% (58–100%)	85% (61–99%)	85% (60–99%)
Chemotherapy			
Dispensed chemotherapy, n	1170 (74%)	3342 (87%)	4512 (83%)
Median number of unique chemotherapies dispensed per person, n (IQR)	1 (0–2)	2 (1–3)	2 (1–3)
Median time on all chemotherapy,^b^ months (IQR)	5.6 (3.3–10.8)	8.3 (4.5–16.5)	7.4 (4.2–15.1)
Median proportion of survival time on chemotherapy, % (IQR)	30% (13–53%)	38% (18–62%)	36% (16–60%)
Endocrine therapy			
Dispensed endocrine therapy, n^d^	885 (56%)	2179 (57%)	3064 (57%)
Median time on all endocrine therapy,^b,d^ months (IQR)	14.5 (3.8–41.2)	16.4 (4.9–43.6)	15.8 (4.6–42.6)
Median proportion of survival time on endocrine therapy, % (IQR)	60% (27–88%)	49% (20–80%)	52% (21–82%)

^a^A course is defined as consecutive dispensings of trastuzumab closer in time than 90 days. A gap of ≥ 90 days between trastuzumab dispensings was considered a break in trastuzumab therapy and the subsequent dispensing considered the start of a new course of therapy.

^b^Excluding breaks in treatment ≥ 90 days

^c^Observed duration of treatment, excluding breaks, as a proportion of observed survival time from initiation of trastuzumab for MBC

^d^Only endocrine therapies dispensed between 30 days prior to initiating trastuzumab for MBC and death or censor

**Table 3:** First-line treatments and cardiac monitoring

	Older patients (≥ 65)	Younger patients (< 65)	Overall
Patients, n	1583	3821	5404
Number of patients initiating trastuzumab with^a^:			
Trastuzumab monotherapy	372 (23%)	637 (17%)	1009 (19%)
Taxane and taxane combinations:	787 (50%)	2368 (62%)	3155 (58%)
Endocrine therapy alone	292 (18%)	490 (13%)	782 (14%)
Vinorelbine	19 (1%)	60 (2%)	79 (2%)
Capecitabine	40 (3%)	58 (2%)	98 (2%)
Other chemotherapy combinations	73 (5%)	208 (5%)	281 (5%)
Patients with a baseline cardiac assessment, n	782 (49%)	1830 (48%)	2612 (48%)
Patients with a cardiac assessment while on trastuzumab, n	956 (60%)	2550 (67%)	3506 (65%)
Patients with a cardiac assessment at baseline and while on trastuzumab, n	607 (38%)	1519 (40%)	2216 (39%)
Number of cardiac assessments perpatient, median (IQR)	4 (2–7)	5 (2–8)	4 (2–8)

^a^First-line partner therapy is based on treatments dispensed during the period from 30 days prior to 90 days following trastuzumab initiation

Other HER2-targeted therapies were dispensed to 340 older patients (21%): 91 (6%) received pertuzumab, 182 (11%) received lapatinib, and 101 (6%) received T-DM1 (Table 2). The median time on all HER2-targeted therapies, including trastuzumab, was 15.4 months (IQR: 6.3–37.0) and the median proportion of survival time spent on HER2-targeted therapies was 85% (IQR: 58–100%; Table 2).

### Chemotherapy and endocrine therapy

Chemotherapy was dispensed to 1170 (74%) older patients and most of these patients received just one chemotherapy medicine (the median number of chemotherapies was 1 (IQR: 0–2; Table 2). The median time on all chemotherapy, excluding breaks in treatment, was 5.6 months (IQR: 3.3–10.8). Most older patients received just one course of chemotherapy (67%), while 13% received three or more courses of chemotherapy. Just over half (58%) of older patients initiated trastuzumab with a chemotherapy partner, most commonly a taxane (50%), and the proportion of patients initiating trastuzumab with chemotherapy increased slightly over the study period (Additional file 1: Figure S1C). Endocrine therapy was dispensed to 885 older patients (56%) for a median duration of 14.5 months (IQR: 3.8–41.2; Table 2). One third of these patients initiated trastuzumab with an endocrine therapy alone (18% of all older patients) while the remaining patients started endocrine therapy after trastuzumab initiation.

### Cardiac monitoring

At least one cardiac monitoring test was undertaken in 1228 (76%) older patients: 782 (49%) had a baseline test (60 days prior to 30 days after starting trastuzumab for HER2+ MBC), 956 (60%) had a test while receiving trastuzumab; 607 (38%) had a test in both time periods (Table 3).

### Overall survival

**Fig. 2:**
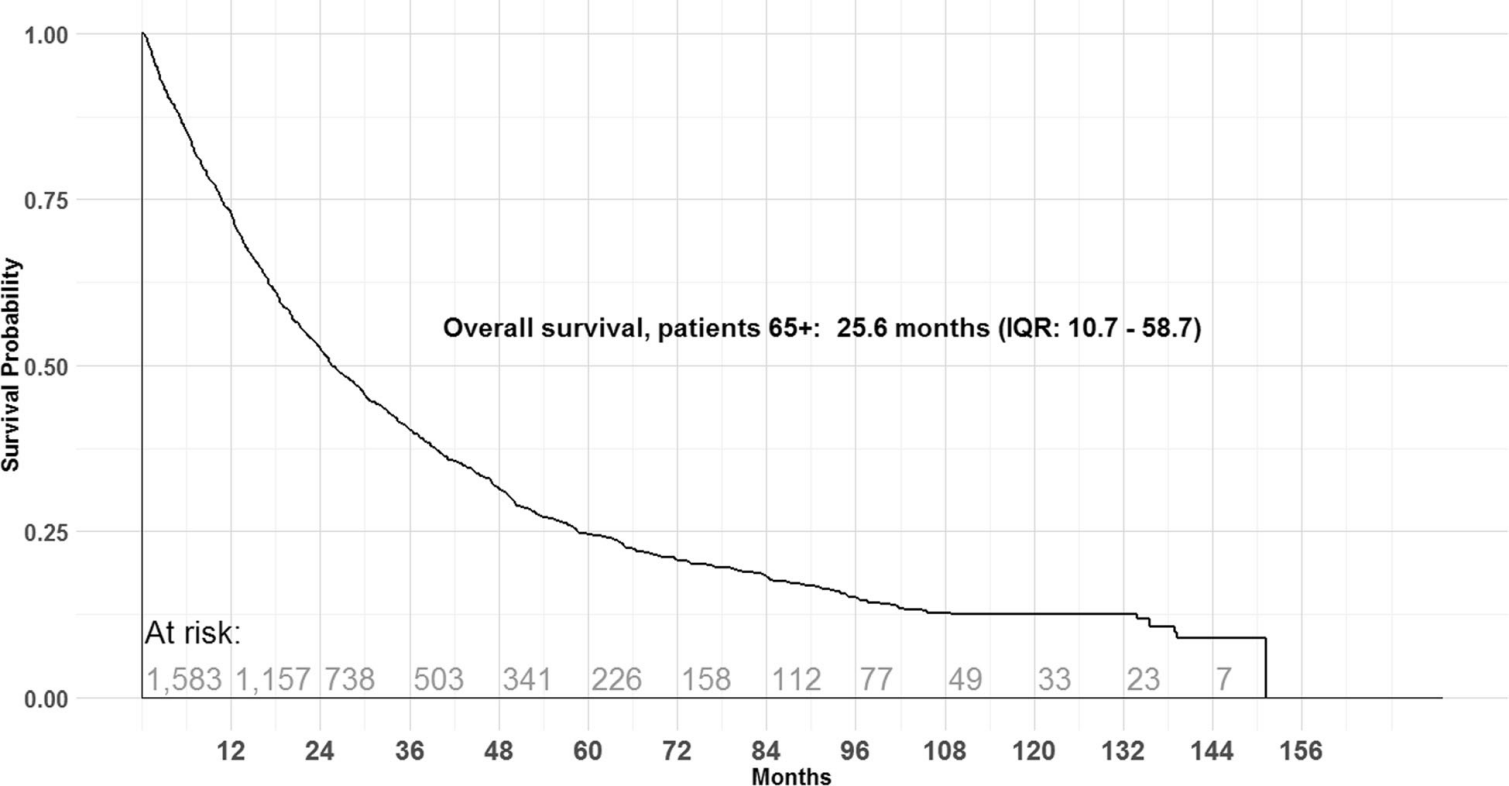
Kaplan-Meier survival probability plot for patients ≥ 65

**Fig. 3:**
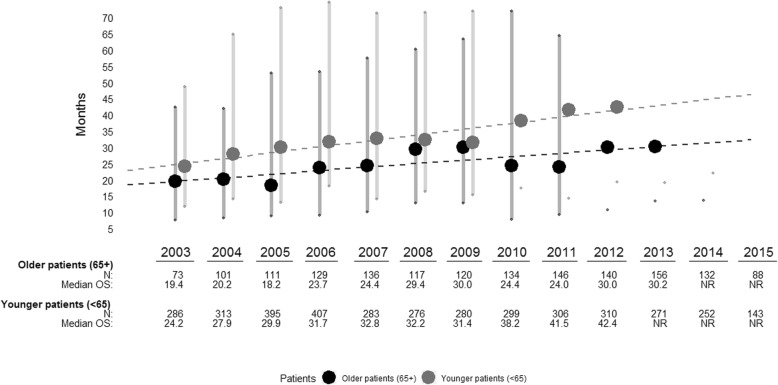
Median overall survival (OS; large dots) and interquartile range (smaller dots and shaded bars) by year of trastuzumab for MBC initiation. The dotted lines indicate the trend in median OS over time. NR = median not reached

By 30 June 2016, 73% of older patients had died. Median OS from initiation of trastuzumab for MBC was 25.6 months (IQR 10.7–58.7; Fig. 2). The median survival for older patients increased with each year of initiation between 2003 and 2015 (Fig. 3), but the increasing trend in median survival was limited to patients who were between ages 65 and 74 at initiation. Median survival for patients ≥ 75 at initiation did not increase over the study period (Additional file 1: Figure S1A). Median survival at initiation was 29.9 months (IQR: 13.3–69.4) for patients aged 65–74 and 19.6 months (IQR: 7.4–47.4) for patients ≥ 75 at initiation (Additional file 1: Figure S1D).

## Discussion

In this population-based cohort study we described the largest cohort of older patients with HER2 + MBC, treated with trastuzumab, in the literature to date. There remains a lack of data about the treatment and outcomes experienced by older patients with HER2 + MBC and our study provides some clarity for these patients. We found that almost a third of all women initiating trastuzumab for MBC between 2003 and 2015 were ≥ 65, with nearly as many women ≥ 75 initiating treatment as those aged 35 to 44. These proportions are substantially larger than those reported in clinical trials, where around 15% of patients were ≥ 65 and less than 2% of patients were ≥ 75 [9, 12]. Moreover, we found that the proportion of older patients treated with trastuzumab for MBC had roughly doubled between 2003 and 2015—from 20 to 38%—but the median age of trial participants has only increased slightly (51 to 54 years) between the original pivotal trial of trastuzumab for MBC and the more recent CLEOPATRA and MARIANNE trials [7, 11, 12]. These findings highlight that older patients are still underrepresented in clinical trials of HER2-targeted agents.

The median duration of the first course of trastuzumab for older patients (12.1 months) was similar to median estimates reported for patients ≥ 65 in the control arms of the CLEOPATRA and MARIANNE trials (trastuzumab plus taxane; 10.4 and 14.6 months, respectively) [9, 12]. We also observed a similar proportion of older patients with previous (neo) adjuvant trastuzumab therapy (12%) as reported from the CLEOPATRA trial (11%) [9], however, (neo) adjuvant trastuzumab was not publicly subsidised in Australia until October 2006 [15], and many patients in our cohort would not have had access to that treatment. The most recent HER2-targeted therapies—pertuzumab and T-DM1—only became subsidized in Australia from July 2015, so few older patients in our study received either medicine.

Survival estimates for patients ≥ 65 in the CLEOPATRA and MARIANNE trials were not reported, however, sub-group analysis of patients > 60 years from the original pivotal trial reported a median OS of 24 months, which is similar to our estimate of 25.6 months. We observed that survival estimates for older patients increased slightly over the study period, primarily in patients initiating trastuzumab for MBC between the ages of 65–74 (Additional file 1: Figure S1A). A similar finding has been reported from studies in wider MBC populations irrespective of HER2-status [21–23], but not specifically in HER2 + MBC cohorts. Our survival estimates for older patients also indicated that nearly one quarter of older patients would survive at least 5 years from trastuzumab initiation for MBC. We have previously reported a similar rate of 5-year survival for trastuzumab-treated HER2 + MBC patients of all ages [19].

There are few observational studies examining the treatment and outcomes of older patients with HER2 + MBC. A handful of studies utilizing hospital-based cohorts have reported proportions as high as 28 and 14% for patients ≥ 65 and ≥ 75 years, respectively [24, 25]. These studies have found that patients ≥ 65 years received less chemotherapy compared to younger patients; had lower median OS; had higher underlying cardiovascular conditions at treatment initiation; and experienced higher rates of cardiotoxic events compared to younger patients [24, 25]. Jackisch et al. also reported that the proportion of older patients in their cohort of women with HER2+ MBC steadily increased during the 10 years of their study period [24].

Our study lacks detailed clinical data and we are unable to control for important patient and disease factors to allow robust, direct comparisons between the older and younger patients in our cohort. However, our findings align with those from previous studies in that we observed that a smaller proportion of older patients were dispensed chemotherapy, and a larger proportion of older patients received trastuzumab as monotherapy (no chemotherapy or endocrine therapy). Similar to Jackisch et al., we found that 18% of older patients and 13% of younger patients initiated trastuzumab with endocrine therapy alone.

Despite the known cardiac risks of trastuzumab treatment, and the fact that older patients are more likely to have comorbidities that increase their risk of cardiotoxicity [10, 25, 26], we observed that only half of the older patients in our cohort received a baseline cardiac assessment (49%), and just over half (60%) received an assessment while on treatment with trastuzumab. MBS data do not include claims for cardiac monitoring tests administered to public hospital inpatients and patients paying for the test out of pocket and it is possible that we do not observe cardiac assessments for some patients. However, previous work has estimated that the MBS captures at least 80% of cardiac monitoring tests [27]. Cardiac assessment was a prerequisite for enrolment in the *Herceptin Program* [15] but this finding suggests that many older patients did not receive this care. The findings were similar in patients < 65 in our cohort where 48% received a baseline cardiac assessment.

As mentioned previously, the primary limitation of our study is a lack of clinical and pathological details. Consequently, we were unable to examine important clinical issues such treatment beyond disease progression and patient outcomes according to metastasis site, hormone receptor status, performance status, and comorbidities. The strengths of this study include the large size and the representativeness of sample drawn from the Australian population. The lengthy, 12.5 years of observation time allowed us to examine trends over time. Our cohort was selected from all women treated with publicly-funded trastuzumab for HER2 + MBC in Australia, which, given the high cost of trastuzumab, likely represents all Australian women treated during the study period. We do not have data for older women diagnosed with HER2 + MBC who did not receive trastuzumab and we cannot examine differences in survival or patient characterists between trastuzumab-treated and untreated HER2 + MBC patients. Furthermore, our data were collected for the primary purpose of reimbursement and they lack of clinical information. We are unable to describe the clinical and comorbidity characteristics of our older patients and how these factors might differ from younger patients initiating trastuzumab during the study period. We do not have diagnoses data and cannot determine which patients may have been diagnosed with early breast cancer; dates of disease progression; and dates of cardiac and other adverse events.

## Conclusions

Our results provide generalizable estimates of time on treatment, OS, and the proportion of patients initiating HER2-targeted therapy from age 65. HER2 + MBC patients are living longer, and it is reassuring that approximately 25% of older patients starting trastuzumab for HER2+ MBC are living longer than 5 years. The survival gains from trastuzumab in older patients may not be as great as those in younger patients, possibly due to increased comorbidities and less administration of chemotherapy. Pertuzumab and T-DM1 can further improve survival outcomes without the toxicity of chemotherapy and these treatments may significantly impact on the survival of older patients. Future population cohort studies on the use of these newer agents in older patients will be useful. Our results highlight the fact that older patients are receiving HER2-targeted therapy in proportions larger than those seen in clinical trials and this proportion is growing. Policy makers will need to take this into account when making subsidy decisions for new targeted therapies.

## Supplementary information

**Additional file 1.** Additional figures showing overall survival stratified by year of trastuzumab initiation and age group (65–74, 75+); proportion of patients initiating trastuzumab for MBC in each year, stratified by age group (< 65, 65–74, 75+); proportion of patients initiating trastuzumab with chemotherapy in each year, stratified by age group (< 65, ≥ 65); and Kaplan-Meier survival probability plot for patients aged 65–74 and patients aged ≥ 75. (PDF 174 kb)

## Data Availability

Details of the source data and record linkage for this study are available in the study protocol [15]. Access to the datasets analysed for the current study is not permitted without the express permission of the approving Human Research Ethics Committee and the data custodian (DHS). Third parties can contact the DHS (https://www.humanservices.gov.au/, statistics@humanservices.gov.au) regarding access to the source data and data custodian approval.

## Abbreviations

AUD: Australian dollar
CLEOPATRA: Clinical Evaluation of Pertuzumab and Trastuzumab, phase III randomised controlled trial
DHS: Department of Human Services
EBC: Early breast cancer
HER2: Human epidermal growth factor 2 gene
HER2+MBC: Human epidermal growth factor 2 gene-positive metastatic breast cancer
IHC: Immunohistochemistry
IQR: Inter-quartile range
ISH: In-situ hybridisation
MARIANNE: A Randomized, 3 Arm, Multicenter, Phase III Study to Evaluate the Efficacy and the Safety of T-DM1 Combined With Pertuzumab or T-DM1 Combined With Pertuzumab-Placebo (Blinded for Pertuzumab), Versus the Combination of Trastuzumab Plus Taxane, as First Line Treatment in HER2 Positive Progressive or Recurrent Locally Advanced or Metastatic Breast Cancer
MBS: Medicare Benefits Schedule
MBC: Metastatic breast cancer
OS: Overall survival
PBS: Pharmaceutical Benefits Scheme
PFS: Progression-free survival
RCT: randomised controlled trial
T-DM1: Trastuzumab emtansine
