# Supplementary material for: Trastuzumab use in older patients with HER2-positive metastatic breast cancer: outcomes and treatment patterns in a whole-of-population Australian cohort (2003–2015)
Source: BMC Cancer. 2019 Sep 11;19:909. doi: 10.1186/s12885-019-6126-y (PMC6740010; doi:10.1186/s12885-019-6126-y)
Supplement: Supplementary file 1 — Additional file 1. Additional figures showing overall survival stratified by year of trastuzumab initiation and age group (65–74, 75+); proportion of patients initiating trastuzumab for MBC in each year, stratified by age group (< 65, 65–74, 75+); proportion of patients initiating trastuzumab with chemotherapy in each year, stratified by age group (< 65, ≥ 65); and Kaplan-Meier survival probability plot for patients aged 65–74 and patients aged ≥ 75. (PDF 174 kb) [file 12885_2019_6126_MOESM1_ESM.pdf]

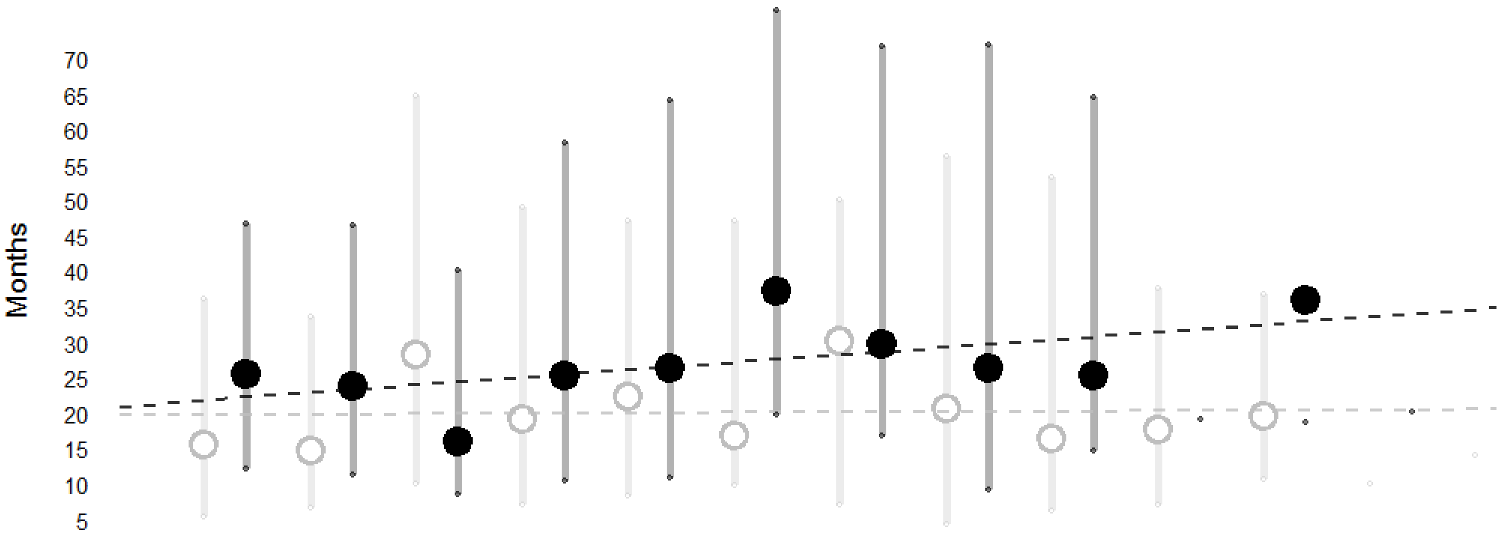

	2003	2004	2005	2006	2007	2008	2009	2010	2011	2012	2013	2014	2015
**Patients 65 - 74**													
N:	41	52	65	71	85	72	77	90	87	75	88	73	60
Median OS:	25.5	23.8	15.9	25.2	26.3	37.1	29.8	26.3	25.2	NR	35.9	NR	NR
**Patients 75+**													
N:	32	49	46	58	51	44	43	44	59	65	68	59	28
Median OS:	15.6	14.7	28.3	19.0	22.2	16.7	30.2	20.5	16.3	17.6	19.6	NR	NR

Figure S1A. Median overall survival (OS; large dots) and interquartile range (smaller dots and shaded bars) by year of trastuzumab for MBC initiation for patients aged 65 – 74 and patients ≥75. The dotted lines indicate the trend in median OS over time. NR = median not reached

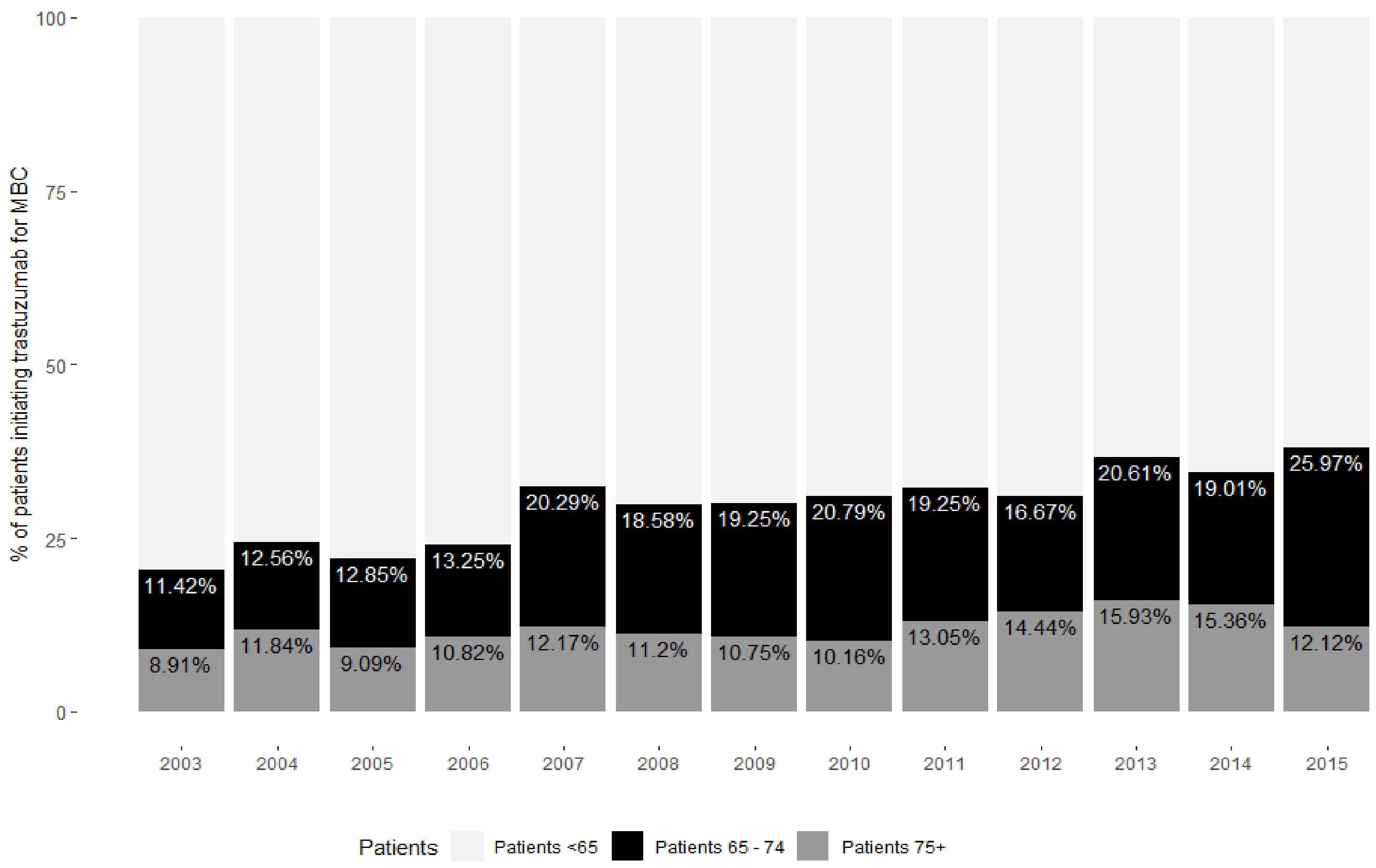

Figure S1B. Proportion of patients initiating trastuzumab for MBC in each year, stratified by age group.

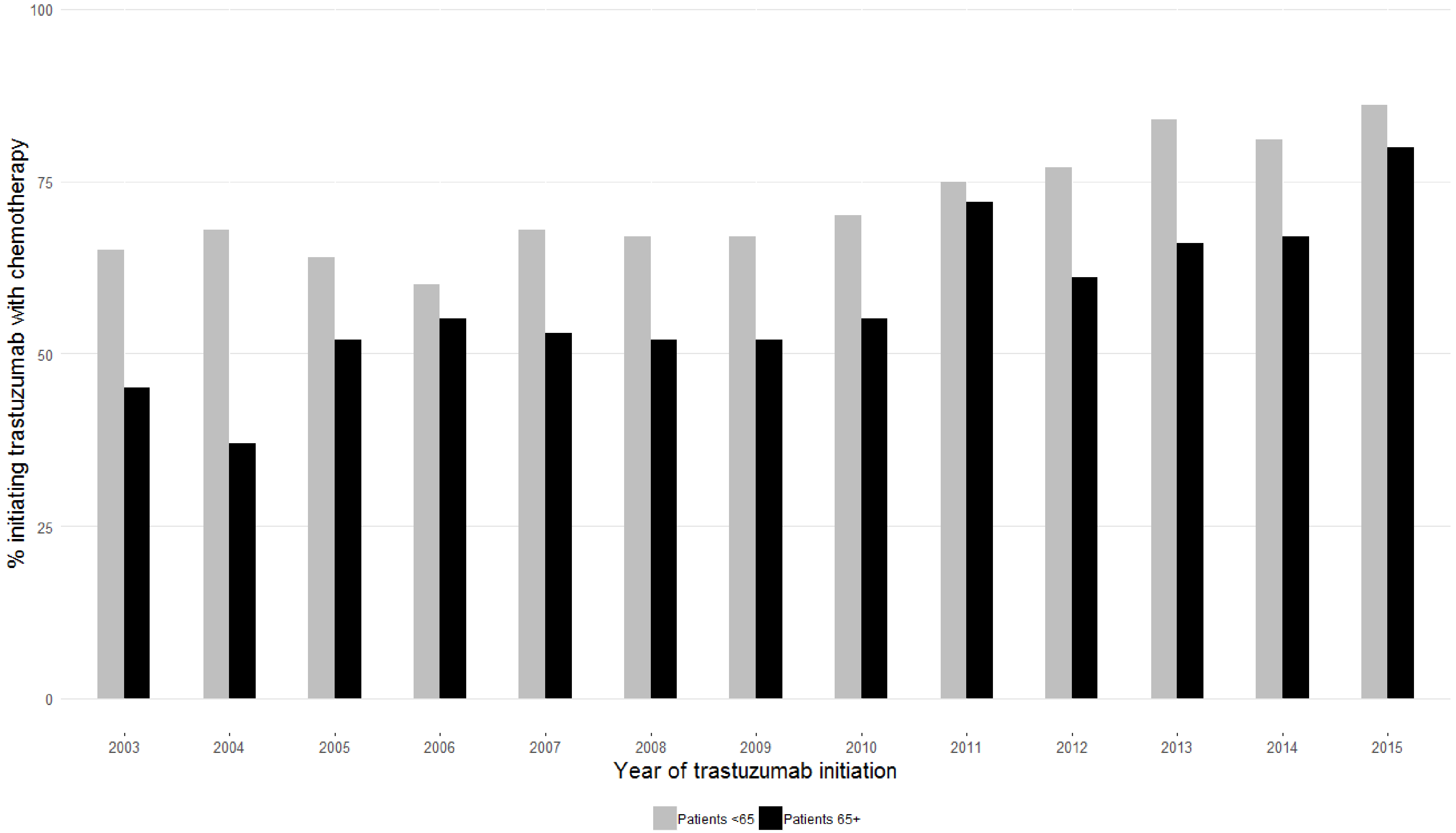

Figure S1C. Proportion of patients initiating trastuzumab with a chemotherapy partner in each year of the study period.

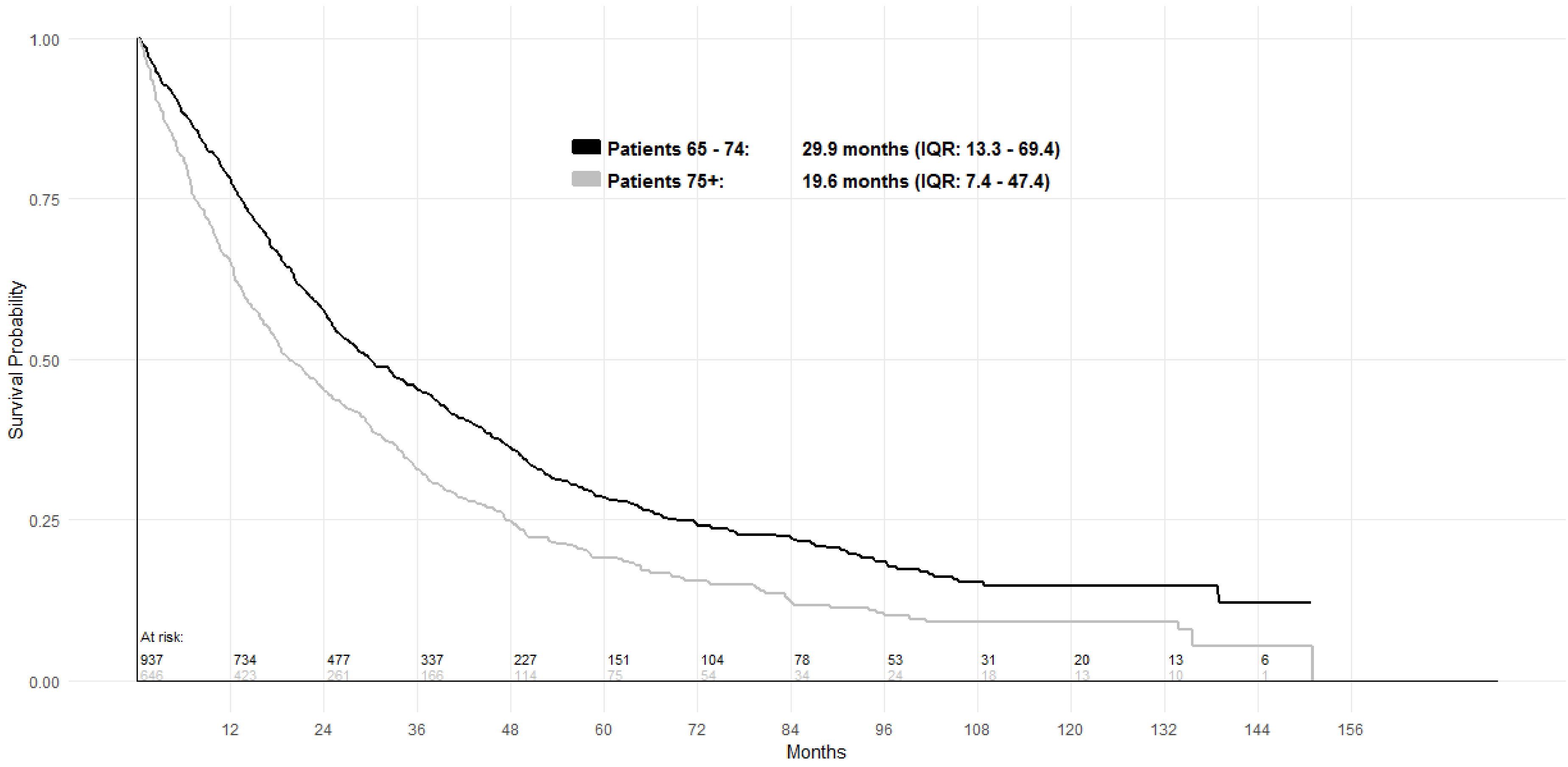

Figure S1D. Kaplan-Meier survival probability plot for patients aged 65 – 74 and patients aged >75.
